# Editorial: Critical care cardiology for cardiovascular emergencies

**DOI:** 10.3389/fcvm.2025.1668820

**Published:** 2025-08-14

**Authors:** Takahiro Nakashima, Keita Saku

**Affiliations:** ^1^ Department of Emergency Medicine and the Harry Max Weil Institute for Critical Care Research and Innovation, University of Michigan, Ann Arbor, MI, United States; ^2^ Department of Cardiovascular Medicine, Graduate School of Medical Sciences, Kumamoto University, Kumamoto, Japan; ^3^ Department of Cardiovascular Dynamics, National Cerebral and Cardiovascular Center, Suita, Japan

**Keywords:** critical care cardiology, veno-arterial extracorporeal membrane oxygenation (V-A ECMO), intra-aortic balloon pump (IABP), microaxial flow-pumps, left ventricular assist device (LVAD), mechanical circulatory support (MCS)

**Editorial on the Research Topic**
Critical care cardiology for cardiovascular emergencies

In recent years, substantial evidence regarding cardiogenic shock and mechanical circulatory support (MCS) has been accumulated in the field of Critical Care Cardiology, contributing to the development of this emerging subspecialty (1). The patient population in cardiac care units (CCUs) has become increasingly complex and critically ill, raising concerns about whether these units can continue to manage high-acuity patients in the absence of intensivists (2). The necessity of critical care cardiologists has been acknowledged for over two decades. However, expert consensus statements on cardiogenic shock have only recently been published by professional societies in Europe, North America, and Japan (3–5). Given the heterogeneity of cardiogenic shock, which encompasses a variety of underlying etiologies, establishing it as a distinct academic field and generating robust evidence has remained a significant challenge. This special issue presents 18 contributions in the realm of critical care cardiology, including 9 original research articles, 3 reviews, 4 case reports, 1 brief research report, and 1 perspective piece (URL: https://www.frontiersin.org/research-topics/65741/critical-care-cardiology-for-cardiovascular-emergencies).

The growing use of LV-unloading devices has heightened the demand for noninvasive methods to assess left ventricular (LV) workload. Sato et al. evaluated the utility of the pressure-strain product (PSP), derived from echocardiographic strain and blood pressure, for estimating pressure-volume (PV) loop-based LV stroke work (LVSW) in a large animal model with a left ventricular assist device (LVAD). PSP demonstrated the strongest correlation with PV loop-derived LVSW, outperforming other parameters such as Echo-derived LV end-diastolic volume, Echo-LVSW, peak LV pressure, and global circumferential strain. Furthermore, PSP significantly correlated with LV myocardial oxygen consumption, pressure-volume area, coronary sinus oxygen saturation, and coronary vascular resistance. Although further clinical studies are warranted, PSP holds promise as a noninvasive biomarker for myocardial metabolic monitoring.

Right heart failure (RHF) following LVAD implantation remains a major clinical concern due to complex hemodynamic interactions that complicate its management. Nonaka et al. provided a comprehensive review of the current understanding of RHF pathophysiology and evaluated existing predictive models for RHF after LVAD placement. The authors' discussion of current knowledge gaps is particularly valuable for improving future RHF management strategies. This review is expected to deepen readers' comprehension of RHF mechanisms.

With the increasing use of MCS, bedside explantation techniques are gaining attention. Xu et al. investigated the feasibility of a novel area-reduction post-closure technique for bedside explantation of veno-arterial extracorporeal membrane oxygenation (V-A ECMO). In their retrospective analysis of 18 patients, the procedure achieved a 100% technical success rate. The authors detailed the method, which notably included the use of an 8 Fr sheath to facilitate the deployment of the first Proglide device—an innovative and immediately applicable technique for clinical practice.

The 2025 ACC/AHA guidelines for acute coronary syndrome give a Class III recommendation against the routine use of intra-aortic balloon pump (IABP) in patients with myocardial infarction complicated by cardiogenic shock. However, the role of IABP remains controversial. Ota et al. presented an intriguing case highlighting the potential importance of aortic compliance in determining IABP efficacy. Their case involved an elderly patient with severe aortic stenosis and pneumonia who developed refractory cardiogenic shock and experienced a dramatic hemodynamic improvement following IABP insertion.

The use of MCS devices such as V-A ECMO and transvalvular micro-axial flow pumps (mAFP) significantly complicates brain death determination and organ donation procedures. Raimann and Willems addressed this challenging issue in patients receiving combined V-A ECMO and tMAFP support.

This issue also includes several additional studies offering valuable insights for clinicians engaged in critical care cardiology. We hope that critical care cardiologists worldwide will take the opportunity to explore these important contributions. As CCUs in developed countries evolve into cardiac intensive care units (CICUs), we are witnessing the dawn of the Critical Care Cardiology era. We look forward to continued advancements in the field, the generation of high-quality evidence, and, ultimately, improved outcomes for critically ill cardiovascular patients.
